# Plant genome editing made easy: targeted mutagenesis in model and crop plants using the CRISPR/Cas system

**DOI:** 10.1186/1746-4811-9-39

**Published:** 2013-10-11

**Authors:** Khaoula Belhaj, Angela Chaparro-Garcia, Sophien Kamoun, Vladimir Nekrasov

**Affiliations:** 1 The Sainsbury Laboratory, Norwich Research Park, Norwich, UK

**Keywords:** CRISPR, Cas9, Plant, Genome editing, Genome engineering, Targeted mutagenesis

## Abstract

Targeted genome engineering (also known as genome editing) has emerged as an alternative to classical plant breeding and transgenic (GMO) methods to improve crop plants. Until recently, available tools for introducing site-specific double strand DNA breaks were restricted to zinc finger nucleases (ZFNs) and TAL effector nucleases (TALENs). However, these technologies have not been widely adopted by the plant research community due to complicated design and laborious assembly of specific DNA binding proteins for each target gene. Recently, an easier method has emerged based on the bacterial type II CRISPR (clustered regularly interspaced short palindromic repeats)/Cas (CRISPR-associated) immune system. The CRISPR/Cas system allows targeted cleavage of genomic DNA guided by a customizable small noncoding RNA, resulting in gene modifications by both non-homologous end joining (NHEJ) and homology-directed repair (HDR) mechanisms. In this review we summarize and discuss recent applications of the CRISPR/Cas technology in plants.

## Introduction

Targeted genome engineering has emerged as an alternative to classical plant breeding and transgenic (GMO) methods to improve crop plants and ensure sustainable food production. However, until recently the available methods have proven cumbersome. Both zinc finger nucleases (ZFNs) and TAL effector nucleases (TALENs) can be used to mutagenize genomes at specific loci, but these systems require two different DNA binding proteins flanking a sequence of interest, each with a C-terminal FokI nuclease module. As a result these methods have not been widely adopted by the plant research community. Earlier this year, a new method based on the bacterial CRISPR (clustered regularly interspaced short palindromic repeats)/Cas (CRISPR-associated) type II prokaryotic adaptive immune system [1] has emerged as an alternative method for genome engineering. The ability to reprogram CRISPR/Cas endonuclease specificity using customizable small noncoding RNAs has set the stage for novel genome editing applications [2-8]. The system is based on the Cas9 nuclease and an engineered single guide RNA (sgRNA) that specifies a targeted nucleic acid sequence. Given that only a single RNA is required to generate target specificity, the CRISPR/Cas system promises to be more easily applicable to genome engineering than ZFNs and TALENs.

Recently, eight reports describing the first applications of the Cas9/sgRNA system to plants have been published [9-16]. In this review, we summarise the methods and findings described in these publications and provide an outlook for the application of the CRISPR/Cas system as a genome engineering tool in plants.

### Plant genome editing using the CRISPR/Cas system

**Figure 1:**
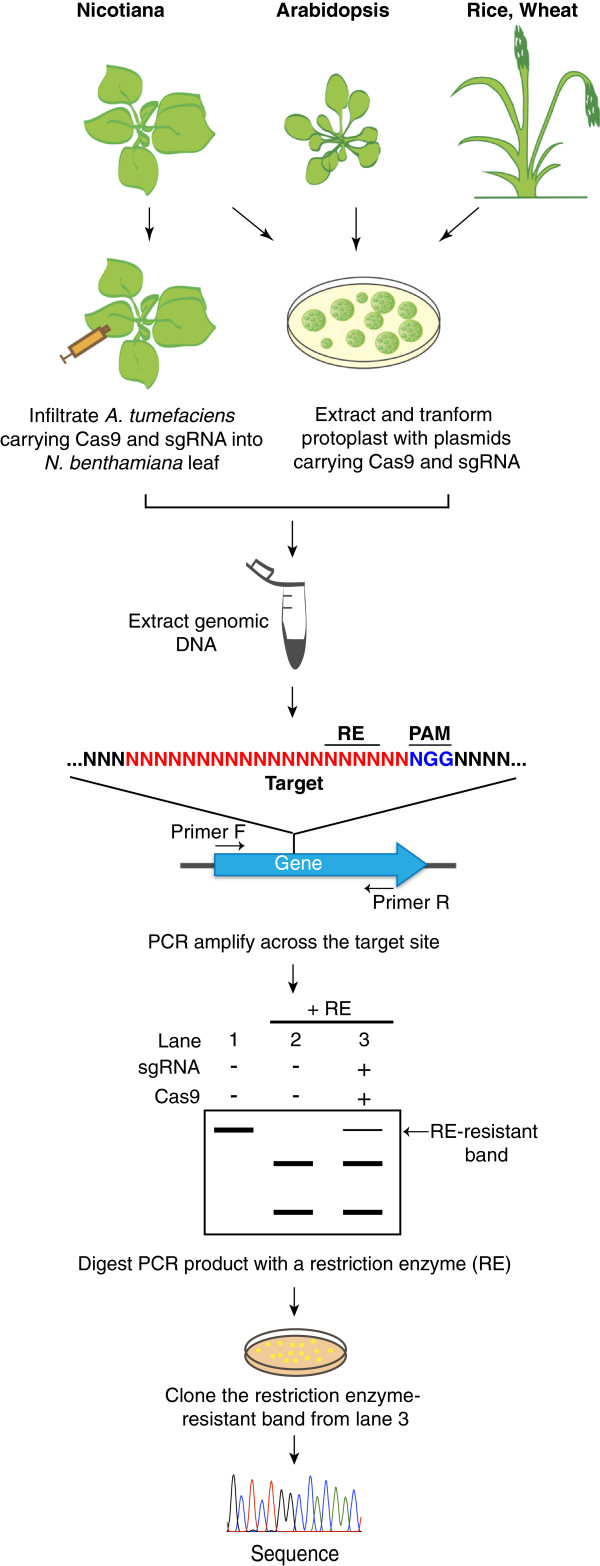
**Schematic drawing illustrating examples of genome editing assays in plants.** The CRISPR/Cas9 technology was successfully applied in model plants (*Nicotiana benthamiana*, *Arabidopsis thaliana*) and crops (rice, wheat). The Cas9 nuclease and the sgRNA matching the gene of interest are co-expressed using *Agrobacterium tumefaciens* as a vector in *N. benthamiana* leaves or transfected into protoplasts from Arabidopsis, wheat or rice. Then, the genomic DNA is extracted from the leaf tissues or protoplasts and subject to PCR-amplification with primers flanking the target site. The presence of Cas9/sgRNA-induced mutations can be easily detected using the restriction enzyme (RE) site loss method. The RE-resistant band (lane 3) can be cloned. The exact nature of the mutations is then revealed by sequencing individual clones.

The application of the bacterial CRISPR/Cas system to plants is very recent. In the August 2013 issue of Nature Biotechnology three short reports described the first applications of the Cas9/sgRNA system to plant genome engineering [9-11]. Shortly after, five more reports followed [12-16]. The papers mainly focused on testing the CRISPR/Cas technology using transient expression assays (Table 1 and Figure 1), such as protoplast transformation and *in planta* expression using *Agrobacterium tumefaciens* transient expression (agroinfiltration) [17]. Mutations introduced via both nonhomologous end joining (NHEJ) and homology-directed repair (HDR) pathways have been reported. Five of the studies generated whole plants that carry mutations at the targeted loci (Table 1).

**Table 1:** Summary of CRISPR/Cas genome editing assays in plants

**Material/activity**	***A. thaliana***	***N. benthamiana***	***O. sativa***	***T. aestivum***	***S. bicolor***	**Reference**
**Cas9**						
Codon-optimized for plants	Yes	Yes	Yes	Yes	Yes	[10,11,15,16]
	No	No	No			[9,12-14,16]
Number of NLS	2	2	2	2		[10-14]
		1				[9,16]
	1		1		N/A	[16]
			1			[7,8,15]
Intron introduced	Yes	Yes				[11]
	No	No	No	No	No	[9,10,12-16]
Promoter	35S PPDK	35S PPDK				[11]
	2x CaMV 35S		2x CaMV 35S	2x CaMV 35S		[10,12]
	CaMV 35S	CaMV 35S	CaMV 35S			[9,11,14,16]
					OsAct1	[16]
	AtUBQ		OsUBQ			[13]
			ZmUBQ			[15]
**sgRNA**						
Promoter	AtU6	AtU6				[9,11-13,16]
			OsU3			[10,12-15]
			OsU6		OsU6	[16]
				TaU6		[10]
**Assays**						
	*AtPDS3*		*OsPDS*			
	*AtFLS2*		*OsBADH2*			
	*AtBRI1*		*OsMPK2*			
	*AtJAZ1*		*Os02g23823*			
	*AtGAI*		*OsROC5*			
	*AtCHL1*		*OsSPP*			
Genes targeted	*AtCHL2*	*NbPDS*	*OsYSA*	*TaMLO*	N/A	[9-16]
	*At5g13930*		*OsMYB1*			
			*OsMPK5*			
			*OsCAO1*			
			*OsLAZY1*			
			*OsSWEET11*			
			*OsSWEET14*			
**Transient assays**						
**Protoplasts**	Yes	Yes	Yes	Yes	No	[10-14,16]
NHEJ mutation frequency	1.1-5.6% [11]	37.7-38.5% [11]	14.5-38% [10]	28-29% [10]		
			3-8% [14]			
HDR modification frequency	18.8% [12]*	9% [11]	6.9% [10]			
	42% [13]*					
**Leaf agroinfiltration**	Yes	Yes	No	No	No	[9,11,16]
NHEJ mutation frequency	2.7% [11]	4.8% [11]				
		2.1% [9]				
**Embryo transformation**	No	No	No	No	Yes	[16]
NHEJ mutation frequency					28%	
**Transgenic mutants**						
**Mutated plants recovered**	Yes	Yes	Yes	N/A	N/A	[9,10,12,13,15]
Frequency of modified plants	30-84% [12]	6.7% [9]	4-9.4% [10]			
	76-89% [13]		5-75% [12]			
			50% [13]			
			83-91.6% [15]			
Biallelic mutations recovered	Yes [12,13]	No [9,11]	Yes [10,12,14,15]	N/A	N/A	
**Off-targets**						
Off-target detected	N/A	No [9]	Yes [10,14]	N/A	N/A	[9,10,14]

*In YF-FP assays.

*Abbreviations*: *A. thaliana: Arabidopsis thaliana, N. benthamiana: Nicotiana benthamiana, O. sativa: Oryza sativa, T. aestivum: Triticum aestivum, S. bicolor: Sorghum bicolor.*

### Cas9 nuclease for plant genome editing

Cas9, a hallmark protein of the type II CRISPR-Cas system, is a large monomeric DNA nuclease guided to a DNA target sequence adjacent to the PAM (protospacer adjacent motif) sequence motif by a complex of two noncoding RNAs: CRIPSR RNA (crRNA) and trans-activating crRNA (tracrRNA) [1,2,18]. In August 2012, Jinek *et al*. showed that a synthetic RNA chimera (single guide RNA, or sgRNA) created by fusing crRNA with tracrRNA is functional to a similar level as the crRNA and tracrRNA complex. As a result, the number of components in the CRISPR/Cas system was brought down to two, Cas9 and sgRNA [2].

**Figure 2:**
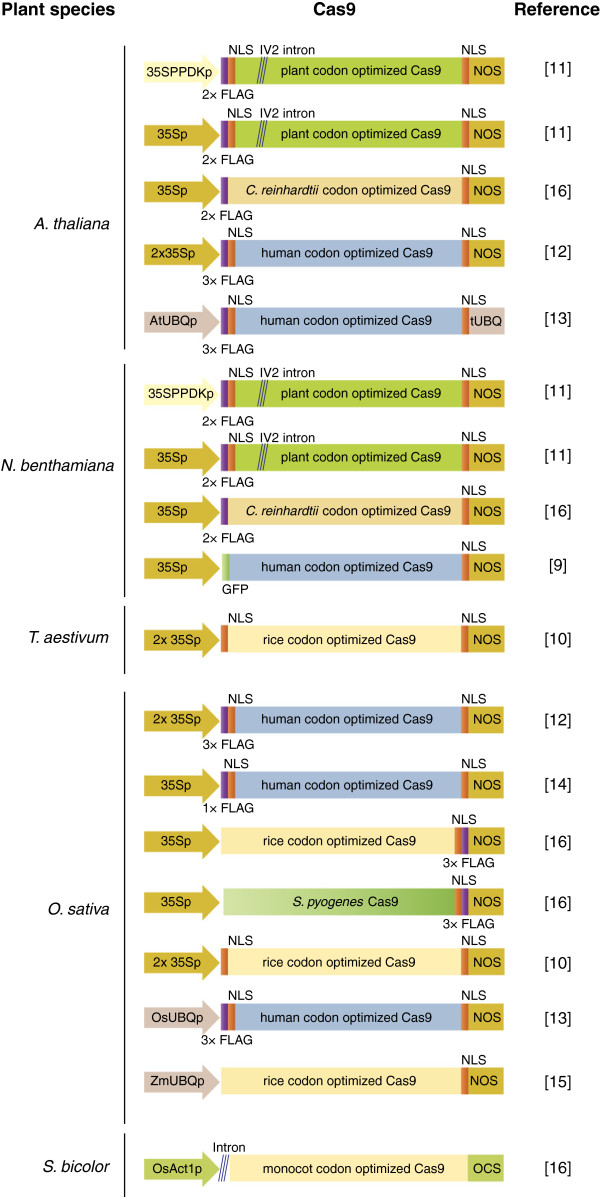
**Cas9 variants used for genome editing in plants.** The Cas9 nuclease was expressed as a fusion protein with a tag (FLAG or GFP as indicated) under various constitutive promoters. Diagonal lines indicate an intron inserted into the *Cas9* gene. NLS, nuclear localization signal.

The Cas9 protein contains two nuclease domains homologous to RuvC and HNH nucleases. The HNH nuclease domain cleaves the complementary DNA strand whereas the RuvC-like domain cleaves the non-complementary strand and, as a result, a blunt cut is introduced in the target DNA [2]. By now, many reports have successfully demonstrated that heterologous expression of Cas9 together with an sgRNA can introduce site-specific double strand breaks (DSBs) into genomic DNA of live cells from various organisms [19]. For applications in eukaryotic organisms, codon optimized versions of Cas9, which is originally from the bacterium *Streptococcus pyogenes*, have been used. Four of the studies on the application of the CRISPR/Cas technology in plants used a plant codon-optimized version of Cas9 [10,11,15,16] while the other four [9,12-14] used the previously described human codon-optimized version (Table 1 and Figure 2). In addition to the codon optimised versions of Cas9, Jiang *et al*. tested the wild type *S. pyogenes* Cas9 and found it was working in rice protoplasts against the *OsSWEET14* target [16].

Li *et al*. introduced an intron into the Cas9 gene (Table 1 and Figure 2) to prevent its expression and avoid toxicity in bacteria but this doesn’t seem to be necessary for delivery by *A. tumefaciens*.

As in the case of human cells [4,5], the Cas9 protein was expressed in plants as a fusion to a nuclear localization signal (NLS) to ensure delivery into nuclei. Cas9 was fused to either a single NLS or was flanked by two NLSs, and, as in human cells, both versions appear to be functional (Table 1 and Figure 2). In addition, six studies used a Cas9 version with a tag (FLAG or GFP), while two studies used a non-tagged Cas9 (Figure 2), suggesting that tagging the protein does not compromise the endonuclease activity *in planta*. Four different promoters were used to drive Cas9 expression with the *Cauliflower mosaic virus* 35S promoter being the most commonly used (Figure 2).

In summary, all tested versions of Cas9 seem to work in plants and very high rates of mutant transgenic plants, generated using the CRISPR/Cas system, have been reported (up to 89% for Arabidopsis and up to 92% for rice) with biallelic mutation being recovered in the case of both plant species (Table 1).

Although the discussed studies provide an insight into functional Cas9 configurations, further studies and side-by-side experiments are required to investigate whether some promoters and Cas9 combinations are more effective than others in plants.

### sgRNAs for plant genome editing

**Figure 3:**
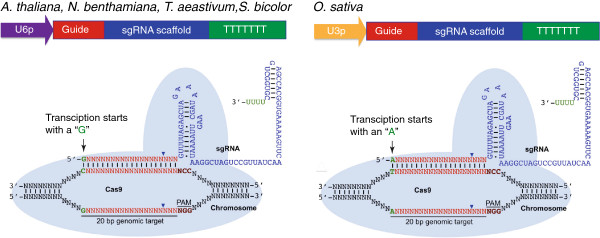
**Scheme illustrating the sgRNA structure and mechanism of the target recognition.** sgRNA is expressed under the U6 promoter in *A. thaliana*, *N. benthamiana, O. sativa*, *T. aestivum* and *S. bicolor*, and under the U3 promoter in *O. sativa*. The transcript initiation nucleotide in the case of U6p and U3p is “G” and “A”, respectively.

The single guide RNA (sgRNA) is the second component of the CRISPR/Cas system that forms a complex with the Cas9 nuclease. As mentioned above, the sgRNA is a synthetic RNA chimera created by fusing crRNA with tracrRNA [2]. The sgRNA guide sequence located at its 5′ end confers DNA target specificity (Figure 3). Therefore, by modifying the guide sequence, it is possible to create sgRNAs with different target specificities. The canonical length of the guide sequence is 20 bp [2]. Consequently, a DNA target is also 20 bp followed by a PAM sequence that follows the consensus NGG (Figure 3). Interestingly, DNA targets and sgRNA guide sequences that differ from the canonical 20 bp length have been reported in some plant studies [10,12-15], while in the mammalian field targets of the consensus (N)_20_NGG are normally used. Therefore, DNA targets validated in plants deviate from the strict (N)_20_NGG and to date follow the consensus (N)_19-22_NGG. The extent to which target sequences that deviate further from this consensus can affect the recognition by the Cas9/sgRNA system remains to be determined.

In plants, sgRNAs have been expressed using plant RNA polymerase III promoters, such as U6 and U3 (Table 1 and Figure 3). These promoters have a defined transcription start nucleotide, which is “G” or “A”, in the case of U6 or U3 promoters, respectively (Figure 3). Therefore, the guide sequences in the sgRNAs, used to target plant genomic loci, follow the consensus G(N)_19–22_ for the U6 promoter and A(N)_19–22_ for the U3 promoter, where the first G or A may or may not pair up with the target DNA sequence [9-16]. On the other hand, in mammalian systems, sgRNA guide sequences normally follow the consensus G(N)_19–20_ where the first G may or may not pair up with the target [20,21].

### CRISPR/Cas genome editing assays in plants

In plants the CRISPR/Cas9 system has been implemented using transient expression systems, therefore enabling rapid execution and optimization of the method. Widely used transient assays in plant research are (i) protoplast transformation and (ii) leaf tissue transformation using the agroinfiltration method. Both methods have been used for Cas9 and sgRNA (Figure 1). The advantage of the protoplast strategy is the possibility to achieve high levels of gene co-expression even from separate plasmids. However, isolation of protoplasts from plant tissue requires enzymatic digestion and removal of the cell wall. The procedure can be time consuming, and protoplast cultures are fragile and prone to contamination. An alternative is the agroinfiltration assay, which is performed on intact plants, and relatively less time consuming compared to protoplasts. This system is based on infiltration of *A. tumefaciens* strains carrying a binary plasmid that contains the candidate genes to be expressed [17]. Efficiency of gene co-expression by agroinfiltration appears to be lower than in protoplasts, and combining multiple genes of interest in one vector is preferable. However, not all plant species are amenable to transformation by these methods and options can be limited depending on the plant species of interest.

To readily detect induced mutations generated by the CRISPR/Cas method, one approach is to target a restriction enzyme site and use the restriction enzyme site loss assay described below (Figure 1). Since the Cas9 nuclease introduces a blunt cut in the DNA predominantly 3 bp away from the PAM (Figure 3), it is advantageous to identify a DNA target with an overlapping restriction site proximal to the PAM motif. In this case, the repair of a DSB via the error-prone NHEJ pathway will result in mutations that will disrupt the restriction site. Therefore, mutations can be detected by amplifying the genomic DNA across the target and digesting resulting amplicons with the restriction enzyme (Figure 1). This assay can be more sensitive when the PCR-amplification is performed on genomic DNA template pre-digested with the restriction enzyme [9,16].

An alternative assay is the Surveyor assay [22]. PCR-amplified DNA from the Cas9/sgRNA treated sample is first denatured and then allowed to anneal before being subject to CELI or T7 endonuclease I that cleave hetero-duplexes formed by the WT and the mutated DNA [13,14]. It is worth considering that the Surveyor assay is less sensitive than the restriction enzyme site loss assay and requires a higher rate of mutagenesis to be successfully applied. However, it can in principle be applied to any target sequence.

**Figure 4:**
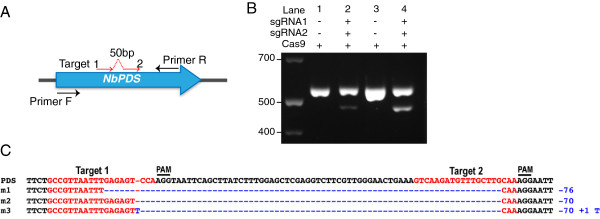
**Generation of a chromosomal deletion by targeting two adjacent sequences within the *PDS *locus of *Nicotiana benthamiana. *A**. Cartoon explaining setup of the experiment. **B**. Detection of deletion mutations using the AFLP analysis. Agarose gel shows PCR bands amplified across targets 1 and 2 using genomic DNA extracted from respective leaf samples. Cas9, sgRNA1 and 2 were expressed in *N. benthamiana* leaf tissue using the standard agroinfiltration protocol. In lane 2, Cas9/sgRNA1/sgRNA2 were expressed from three separate plasmids, while in lane 4 they were expressed from a single plasmid. **C**. Types of deletion mutations identified. Bottom PCR bands from lanes 2 and 4 were cloned into a high copy vector and 15 individual clones were sequenced. All clones contained deletions that can be grouped in three different types (m1-3).

The efficiency of gene mutagenesis can be improved by creating a large deletion. This can be achieved by simultaneously introducing two DSBs guided by two sgRNAs targeting the same locus. For example, a large deletion was introduced by targeting two juxtaposed target sequences on the chromosome in Arabidopsis [11,13]. A similar approach can be implemented in *N. benthamiana* using the agroinfiltration assay to generate targeted deletions (Figure 4; Materials and Methods). Co-expression of Cas9 with sgRNAs, targeting two adjacent sequences 50 bp apart, resulted in a large deletion in the *NbPDS* gene. The AFLP (amplified fragment length polymorphism) assay was used to detect deletions. DNA from the lower PCR band in lanes 2 and 4 (Figure 4B) was cloned and sequenced. Sequencing 15 individual clones revealed presence of 3 types of deletions (Figure 4C). As illustrated by the Figure 4B, the efficiency of the mutagenesis was higher in the case of Cas9 and both sgRNA1 and 2 being expressed from the same plasmid. Transgenic *N. benthamiana* plants can be easily regenerated out of the agroinfiltrated tissue [9] and therefore it should be possible to generate plants carrying the specified deletions.

### Homology-directed repair (HDR) using CRISPR/Cas system in plants

The DSB introduced by Cas9 nuclease guided by an sgRNA can be repaired via either the cell’s NHEJ or HDR mechanisms. NHEJ can be harnessed to generate single and multiple gene knock-outs as well as large chromosomal deletions following cuts generated by CRISPR/Cas. HDR, on the other hand, enables targeted gene insertions (e.g. introducing a green fluorescent protein GFP tag into a genomic locus) or gene replacements (e.g. introducing a SNP into a gene of interest) [22]. HDR-dependent genome editing using the CRISPR system has been achieved in *N. benthamiana*[11] and rice [10]. The donor DNA, which is used as a repair template, was delivered into protoplasts as a single stranded oligo [10] or as a double stranded DNA fragment [11]. The next challenge would be to regenerate whole plants from protoplasts and so far this is only possible for some plant species (e.g. *N. benthamiana* and Arabidopsis).

HDR using CRISPR/Cas system has not yet been achieved in plants using *A. tumefaciens* delivery. In principle, the DNA repair template can be delivered together with the Cas9 and sgRNA in a T-DNA carrying all three components as reported for the I-SceI meganuclease [23]. For plant species that are not amenable to transformation by *A. tumefaciens* and cannot be regenerated out of protoplasts, the Cas9/sgRNA and donor DNA can be delivered into plant cells by callus bombardment as described for cotton in D’Halluin *et al*. [24].

HDR-mediated genome editing can be problematic due to intrinsically low efficiency of homologous recombination (HR) as in the case of Arabidopsis [11]. The NHEJ DNA repair pathway is antagonistic to the HDR pathway. Therefore, HDR efficiency can be increased using mutants compromised in the NHEJ DNA repair mechanism (e.g. *ku70* and *lig4*). In Arabidopsis, an increase of 5–16 fold in HDR-mediated gene targeting has been reported for the *ku70* mutant and 3–4 fold for the *lig4* mutant [25]. Once the desired gene-targeting event is produced, the *ku70* or *lig4* mutations can be crossed out of the mutant plants.

### Off-target mutations and plant genome editing

Target specificity is an important issue for all genome editing technologies, including CRISPR/Cas. Recently, a number of reports have systematically examined specificity of the CRISPR/Cas system in human cells as well as *in vitro*[26-30]. The main conclusion is that the 3′ end of the guide sequence within the sgRNA predominantly confers target specificity of the CRISPR/Cas system. This is consistent with earlier reports [2,5,8]. Mismatches between the DNA target and the guide sequence of the sgRNA located within the last 8–10 bp of the 20 bp target sequence often abolish the target recognition by Cas9, while mismatches towards the 5′ end of the target are better tolerated. Presence of the PAM motif (NGG) right after the 20 bp target is essential, although Hsu *et al*. reported that a variant of the PAM with a noncanonical NAG sequence retains some activity [29]. Importantly, the number and position of tolerable mismatches between the DNA target and the guide sequence is target-dependent and users should be careful not to generalize the reported rates [26,29].

How prone is the CRISPR system to off-target activity when applied in plants? Off-targets were addressed in four reports [9-11,14]. Two of them detected experimental evidence of off-target activity in rice [10,14]. However, Nekrasov *et al*. did not detect off-target activity in *N. benthamiana* for 18 off-sites with sequence similarity to the target [9]. Overall, the number of tested off-sites in all studies was relatively small and general conclusions would be premature. A comprehensive study based on whole genome sequencing of mutant plants is required to fully address this issue *in planta*.

Off-target mutations by the CRISPR system can be minimised by selecting target sequences that have reduced numbers of off-targets in the genome. Examples of algorithms for selecting specific targets have been reported for Arabidopsis and rice [11,14,15]. In any case, off-target mutations are less problematic in plants compared to animals, because they can easily be crossed out.

### Outlook

The CRISPR/Cas technology has enormous potential as a straightforward genome-editing tool for basic and applied plant research. Considering the number of reports that have already been published on plant applications, the method appears to be easily applicable and robust. The major advantage of the CRISPR/Cas technology over ZFNs and TALENs is that the method does not require elaborate design and time-consuming assembly of individual DNA-binding proteins. In contrast, the CRISPR/Cas system is versatile and only requires a single Cas9 nuclease that can be programmed by engineering the sgRNA.

Until recently, the possibility of recovering knockout lines by conventional reverse genetic approaches (T-DNA, TILLING) for a specific gene has been dictated by chance. The shorter the gene, the lower the probability to hit it with a T-DNA insertion or a mutation. Routine targeted mutagenesis opens up a new dimension in plant biology and should help to generate mutants in previously difficult to access genes, as well as simultaneously mutate multiple loci and generate large deletions [11,13]. The likelihood of targeting a specific genomic locus is probably affected by various factors (e.g. chromatin context) but Cas9 does not seem to be affected by DNA methylation, at least in human cells [29].

We foresee the CRISPR technology to become a routine method in plants for making targeted single and multiple gene knock-outs, introducing SNPs into a gene of interest, expressing proteins tagged with affinity or fluorescent tags at their native loci in the genome and much more. However, some questions remain to be addressed as the technology has only been implemented for a few months. One of the outstanding issues is whether genetic changes induced by Cas9/sgRNA can be inherited by the plant germline and transferred to subsequent generations. Genotyping the progeny of plants carrying Cas9/sgRNA induced mutations will answer this question. The relatively high off-target rate of the CRISPR system could be an issue as well. However, off-target effect can be minimised by making an informed decision about the choice of target sequence within a gene according to the algorithms described [11,14,15]. The plant field will soon benefit from an online tool analogous to http://crispr.mit.edu/[29] for designing CRISPR targets with a minimum off-target effect in various plant species. As mentioned earlier, the off-target mutations in plants are less problematic compared to human or animals as they can be easily bred out.

Like ZFNs and TALENs, the CRISPR technology has become one of the new plant breeding techniques (NPBTs). NPBTs are currently debated by advisory and regulatory authorities in Europe and worldwide in relation to the GMO legislation [31-34]. These techniques make possible introducing plant genome modifications, which are indistinguishable from those introduced by conventional breeding and chemical or physical mutagenesis. As a result, crop varieties produced using the above mentioned technologies may be classified as non-GM. Excluding such crop varieties from the scope of the GMO legislation, especially in Europe, would have an enormous positive impact on the development of the plant biotechnology and breeding sector.

## Materials and methods

### Plasmid construction

In the case of the lane 2 (Figure 4B), pK7WGF2::Cas9 and pICH86966::AtU6p::sgRNA_PDS (sgRNA1) [9] were co-expressed with pICH86966::AtU6p::sgRNA2 in *N. benthamiana* using the standard agroinfiltration protocol. pICH86966::AtU6p::sgRNA2 was created in the same way as pICH86966::AtU6p::sgRNA_PDS (sgRNA1) except that the oligos used to construct the sgRNA2 were PDS_gRNA2_BsaIf and gRNA_AGCG_BsaIr (Table 2).

**Table 2:** Primers used in this study

**Primer name**	**Sequence 5′ to 3′**
PDS_gRNA1_BsaIf	TGTGGTCTCAATTGCCGTTAATTTGAGAGTCCAGTTTTAGAGCTAGAAATAGCAAG
PDS_gRNA2_BsaIf	TGTGGTCTCAATTGTCAAGATGTTTGCTTGCAAGTTTTAGAGCTAGAAATAGCAAG
gRNA_AGCG_BsaIr	TGTGGTCTCAAGCGTAATGCCAACTTTGTAC
Cas9_1f	GAGGAAGACAAAATGGACAAGAAGTACTCCATTGGG
Cas9_1r	GAGGAAGACAAAGTCTCTTCTGATTTGCGAGTCATCCA
Cas9_2f	GAGGAAGACAAGACTATCACTCCCTGGAACTTCGAG
Cas9_2r	GAGGAAGACAAATCTTCTTTGAGCTGTTTCACGGTAACT
Cas9_3f	GAGGAAGACAAAGATTATTTCAAAAAGATTGAATG
Cas9_3r	GAGGAAGACAAGAGACTGTCCCCCTGGCCAGAAACTTG
Cas9_4f	GAGGAAGACAATCTCCACGAGCACATCGCTAATCTTGCAGG
Cas9_4r	GAGGAAGACAAGAAACCAGCTTAGACTTCAGAGTAATA
Cas9_5f	GAGGAAGACAATTTCAGATTTCAGAAAGGACTTTCAG
Cas9_5r	GAGGAAGACAAAGATCCTTTGAGCTTTTCATAGTGGCTGG
Cas9_6f	GAGGAAGACAAATCTCCCGAAGATAATGAGCAGAAG
Cas9_6-1r	GAGGAAGACAAAAGCTCACACCTTCCTCTTCTTCTTGG
PDS_MlyIF	GCTTTGCTTGAGAAAAGCTCTC
PDSseqr5	TTTAAAGGATTAAAGTCCTTTGTCA
M13 forward	GTTGTAAAACGACGGCCAGT
M13 reverse	CAGGAAACAGCTATGACC

In the case of the lane 4 (Figure 4B), Cas9 and both sgRNA1 and 2 were expressed in *N. benthamiana* from the single construct pAGM4723::AtU6p::sgRNA2-2x35S-5′UTR::Cas9::NOST-AtU6p::sgRNA1 as described above for the lane 2. The construct was assembled using the Golden Gate (GG) cloning method [35] as following. sgRNA1 was PCR-amplified with primers PDS_gRNA1_BsaIf and gRNA_AGCG_BsaIr, and sgRNA2 – with primers PDS_gRNA2_BsaIf and gRNA_AGCG_BsaIr using the plasmid gRNA_GFP_T1 [4] as a template. Both sgRNA1 and sgRNA2 PCR products were cut-ligated with the AtU6p level 0 module [9] into pICH47751 and pICH47732 level 1 vectors [35] respectively using BsaI.

In order to use the human codon optimised Cas9 [4] in the GG system, all BsaI and BbsI sites had to be removed from its sequence, while preserving the amino acid composition of the protein, in a process called “domestication”. Fragments of the Cas9 coding sequence were amplified with six pairs of primers: Cas9_1f/Cas9_1r, Cas9_2f/Cas9_2r, Cas9_3f/Cas9_3r, Cas9_4f/Cas9_4r, Cas9_5f/Cas9_5r and Cas9_6f/Cas9_6-1r using the clone described in Mali *et al*. as a template. The resulting PCR fragments were cloned into the pCR-Blunt II-TOPO vector (Life Technologies). All six cloned fragments of Cas9 were then cut-ligated into a level 0 vector [35] using BbsI. The resulting Cas9 level 0 module was combined with 2x35S-5′UTR (pICH51288) and NOST (pICH41421) level 0 modules (provided by S. Marillonnet) and cut-ligated into the pICH47742 level 1 vector [35] using BsaI.

pICH47732::AtU6p::sgRNA2, pICH47742::2x35S-5′UTR::Cas9::NOST and pICH47751::AtU6p::sgRNA1 level 1 constructs plus pELE-3 linker [35] were cut-ligated into the pAGM4723 level 2 vector (provided by S. Marillonnet) using BbsI. The resulting level 2 construct pAGM4723::AtU6p::sgRNA2-2x35S-5′UTR::Cas9::NOST-AtU6p::sgRNA1 was transformed into the AGL1 strain of *A. tumifaciens*.

### Transient gene expression in *N. benthamiana*

Transient expression was performed using the AGL1 strain of *A. tumefaciens* as described in Bos *et al*. [36].

### Detection of Cas9-induced deletions in plant genomic DNA

Cas9 and sgRNAs were transiently co-expressed in the *N. benthamiana* leaf tissue*.* The tissue was harvested at 2 days post infiltration and the genomic DNA extracted using the DNeasy Plant Mini kit (Qiagen). 50 ng of DNA was added in a PCR reaction and amplified with PDS_MlyIF and PDSseqr5 primers using the Phusion DNA polymerase (New England Biolabs). PCR products were run on a 3% agarose gel. The DNA from bottom bands in lanes 2 and 4 (Figure 4) was extracted and cloned into pCR-Blunt II-TOPO vector (Life Technologies). 15 individual clones were sequenced using standard M13 forward and M13 reverse primers.

## Competing interests

The authors declared that they have no competing interests.

## Authors’ contributions

SK proposed the idea of the manuscript and wrote the outline. VN performed experiments. KB, ACG, SK and VN wrote the manuscript. All authors read and approved the final manuscript.
